# Validity evidence of the Brazilian version of the Perceived Motivational Climate in Sports Questionnaire-2 (PMCSQ-2BR)

**DOI:** 10.1186/s41155-022-00230-2

**Published:** 2022-09-16

**Authors:** Daniel Perez Arthur, Mayara Juliana Paes, Thais do Amaral Machado, Joice Mara Facco Stefanello

**Affiliations:** Departamento de Educação Física–Universidade Federal do Paraná Avenida Coronel Francisco Heráclito dos Santos, 100 Caixa Postal 19061 Campus Centro Politécnico CEP 81531-980, Curitiba, PR Brazil

**Keywords:** Motivational climate, Psychometrics, Evidences of validity, Team sports, Cross-cultural adaptation, PMCSQ-2

## Abstract

In sports context, the motivational climate has been widely studied since 2000, when the Perceived Motivational Climate in Sports Questionnaire-2 (PMCSQ-2) was published. Evaluating athletes’ perceptions of the motivational climate created by the coach, this questionnaire has been validated and adapted for different countries, including Brazil. However, important psychometric properties of the Brazilian version present problems, such as poor fit indices and the almost exclusively male samples, make it unfeasible for use in future research. Thus, this study aimed to achieve a new process of cross-cultural adaptation, and search for validity evidence of the PMCSQ-2 for the Brazilian sports context to correct distortions, expand the sources of evidence of validity and ecological validity, and make it suitable for application in future research. The sample consisted of 501 athletes (349 males, 152 females) from different sports. The findings of the current study support the multidimensional hierarchical characteristic of the instrument, its factorial structure, and internal consistency. We concluded that the 33-item PMCSQ-2BR, distributed in two high-order scales (ego-involving and task-involving) with three subscales each, can be used to assess athletes’ perceived motivational climate in the Brazilian sports context.

## Introduction

The coach-created motivational climate plays an important role in the development of athletes and sports teams and has been widely studied in past decades. Based on the achievement goal theory (AGT) (Ames, 1992; Archer & Ames, 1988; Nicholls, 1984), two major facets of athletes’ perception of the motivational climate created by coaches were identified in sports contexts: an ego-involving climate and a task-involving climate (Ames, 1992; Nicholls, 1984; Selfriz et al., 1992). An ego-involving climate is perceived, for example, when the coach emphasizes rivalry between members of the same team, is punitive toward his athletes’ mistakes or focuses more attention on the most skilled athletes. However, a task-involving climate is perceived when the coach emphasizes the importance of all athletes to the team and encourages individual improvement and effort by his athletes as well as cooperation among them (Duda & Balaguer, 2007). Studies on the coach-created motivational climate show that athletes from the same team tend to have similar perceptions of the team’s climate (Balaguer et al., 1997; Gano-Overway et al., 2005), suggesting that these two facets could play an important role in the goal orientation adopted by athletes who are exposed to them.

Recent studies have demonstrated the long-term impact of goal orientation on the affective and behavioral aspects of athletes. Evidence shows that athletes with task-oriented goals showed a positive correlation with perceived improvement and satisfaction with their performance level, pleasure in practicing sports, intrinsic motivation, self-confidence, use of positive thoughts, and adoption of behaviors considered desirable in a sports context.⁠ On the other hand, athletes with ego-oriented goals show a positive correlation with less intensity with factors such as perceived performance and motivation and exhibited greater intensity with burnout, anxiety, and adoption of behaviors considered undesirable and unsportsmanlike (Biddle et al., 2003; Lochbaum et al., 2016).

To evaluate this psychological construct, specific instruments must be developed and validated for the population and contexts to be studied. These instruments must provide detailed psychometric characteristics to ensure that they can be applied in a reliable manner to reduce the chances of errors and bias in the research. These characteristics are obtained through several sources of validity evidence, which include evidence based on test content, response processes, internal structure, and relations to other variables (American Educational Research Association, American Psychological Association, National Council on Measurement in Education, 2014).

A commonly used strategy in scientific research is the translation and cross-cultural adaptation of foreign instruments that have already demonstrated good psychometric properties (Beaton et al., 2007). This is important not only because it avoids the creation of a new instrument that measures the same construct as one that is already validated and widespread, but also because the more validations exist in different populations and different contexts of a given instrument, the greater its ecological validity. However, these cross-cultural adaptations and translations should go through the same rigorous processes of obtaining sources of validity evidence as a new instrument because the use of translated questionnaires from other countries and cultures or those that do not present good psychometric properties can lead to a distorted evaluation of what is to be measured. The result may be inaccurate, inconclusive, and unreliable results (Beaton et al., 2000).

The most used instrument to assess athletes’ perception of the motivational climate created by the coach and that has the best psychometric properties is the Perceived Motivational Climate in Sports Questionnaire-2 (PMCSQ-2) (Newton et al., 2000). It contains two high-order scales (ego-involving and task-involving climates), each with three subscales, (punishment for mistakes, unequal recognition, and intrateam member rivalry subscales in the ego-involving scale and cooperative learning, important role, and effort/improvement subscales in the task-involving scale).

In addition to the original version of the questionnaire (Newton et al., 2000), PMCSQ-2 has been translated into different languages, including Spanish (Balaguer et al., 1997), Brazilian Portuguese (Fernandes et al., 2018), Norwegian (Møllerløkken et al., 2017), and Hungarian (Revesz et al., 2014).

Although the PMCSQ-2 and its respective translations are properly based on the AGT, which provides theoretical support to the instrument and has been widely used in research over the past 20 years (Harwood et al., 2015), some fit indices were below expectations in their confirmatory factor analyses. This indicates that some adjustments should be made in the analyses of the instrument. The use of maximum likelihood (ML) as an estimator in their analysis may have contributed to the unsatisfactory results. Widely used in the validation of questionnaires, this method of estimation is applied to continuous scales and is very sensitive to sample size (Brown, 2015). For categorical scales (such as the Likert scale), different estimators, such as weighted least square mean and variance adjusted (WLSMV), may be more appropriate. The current Brazilian version published by Fernandes et al. (2018), despite being an important initiative for the assessment of the motivational climate created by coaches in the Brazilian sports context, presents poor fit indices that are considered insufficient or inadequate to guarantee psychometric rigor for validation. In addition, important methodological limitations, such as the exclusion of items (situations that did not occur in any other foreign validation), and the population sample composed almost exclusively by men, make it unfeasible for unrestricted use without further studies to improve its psychometric properties.

In light of the importance of the PMCSQ-2 for the study of the coach-created motivational climate and the limitations found in its Brazilian version, this study aimed to seek evidence of validity and reliability in the original version of the PMCSQ-2 (Newton et al., 2000), which is translated and cross-culturally adapted for Brazilian athletes participating in the 19th Paraná Open Games and/or the 32nd Paraná Youth Games. The search for validity based on the content of the test, internal structure of the instrument, and its relationship with other variables, in addition to the test-retest reliability, are considered. This study is relevant because the search for evidence of validity with respect to psychometric instruments should be continuous, thus providing professional practice with duly validated and reliable instruments to reduce errors and research bias.

This study introduces statistical tests suggested by the original authors (Newton et al., 2000) that are not covered in the Brazilian version published (Fernandes et al., 2018), includes other tests that may increase the sources of validity of the instrument, and utilizes the WLSMSV estimator in the PMCSQ-2 Confirmatory Factor Analysis (CFA). It is hypothesized that the WLSMV estimator may be more adequate for the factorial planning of the Brazilian version of PMCSQ-2. The task-involving high-order scale and their respective second-order scales will be positively correlated with task-oriented athletes, and the ego-involving high-order scale and their respective second-order scales will be positively correlated with ego-oriented athletes, as suggested by the initial AGT studies (Ames, 1992; Archer & Ames, 1988; Nicholls, 1984).

Another relevant aspect of this study is the adoption of the guidelines proposed by the contemporary version of the Standards for Educational and Psychological Tests (2014), which addresses the growing concern of researchers regarding the process of developing and validating instruments.

## Method

### Participants

#### Translation/back-translation

Four translators (two for translation of English/Portuguese and two for back translation of Portuguese/English), all former athletes and sport science researchers, were invited to participate in this study.

#### Semantic analysis

Thirty-four volleyball athletes who participated in the 18th Paraná Volleyball Cup were recruited. They were aged 12–21 years (mean = 15.15, *s* = 2.41), had 4.59 (*s* = 2.33) years of experience in the sport, 2.62 (*s* = 1.86) years of experience with the same team/coach, 17 (50%) attended elementary school, 10 (29.41%) attended high school, and 7 (20.59%) had already completed high school or were in college.

#### Evidence based on test content

Twelve experts with 17.67 (*s* = 5.93) years of experience in research in sports psychology (100%) and/or psychometry (66.67%) and motivation (100%) were selected through a search conducted in the database of the National Council for Scientific and Technological Development (CNPq).

#### Evidence based on internal structure

A total of 501 athletes from 19 different cites who participated in the 19th Paraná Open Games and/or the 32nd Paraná Youth Games were selected. Of these, 349 (69.66%) were male and 152 (30.34%) female. These participants were aged 13–46 years (mean = 20.59, *s* = 5.90 years); represented five different sports: basketball (*n* = 29), soccer (*n* = 117), futsal (*n* = 111), handball (*n* = 106), volleyball (*n* = 138); had an average of 9.08 (*s* = 5.55) years of experience in the sport; and 2.99 (*s* = 3.76) years of experience with the same coach.

#### Test-retest reliability

Fifty-one athletes from 11 different cities of Paraná State participated in the 19th Paraná Open Games and/or the 32nd Paraná Youth Games, of which 35 (68.43%) were male and 16 (31.37%) were female. They were aged 13–38 years (mean = 22.06, *s* = 5.01 years) and represented four different sports, soccer (*n* = 11), futsal (*n* = 11), handball (*n* = 11), volleyball (*n* = 18), with an average of 10.32 (*s* = 4.16) years of experience in the sport and 2.53 (*s* = 3.29) years of experience with the same coach.

### Evidence based on relations to other variables

#### Discriminant evidence

A total of 224 athletes from 17 different cities of Paraná State participating in the 19th Paraná Open Games and/or the 32nd Paraná Youth Games, were selected, of which 146 (65.18%) were male and 78 (34.82%) were female, aged 16–38 years (mean = 17.98, *s* = 4.46 years); representing five different sports, basketball (*n* = 16), soccer (*n* = 63), futsal (*n* = 46), handball (n = 51), and volleyball (*n* = 48); had an average of 7.17 (*s* = 4.54) years of experience in the sport; and 2.64 (*s* = 2.96) years of experience with the same coach.

### Instruments

The PMCSQ-2 is a questionnaire designed to evaluate athletes’ perception of the motivational climate created by their coach in the sports context. It is a 33-item questionnaire that presents two high-order scales (ego-involving and task-involving) with three subscales each (punishment for mistakes, unequal recognition, and intra-team member rivalry subscales in the ego-involving scale and cooperative learning, important role, and effort/improvement subscales in the task-involving scale), with each item being evaluated using a 5-point Likert scale (1 = strongly disagree, 5 = strongly agree). It presents an internal consistency of α = 0.88 to the task-involving scale, α = 0.87 to ego-involving scale, α = 0.74 to cooperative learning subscale, α = 0.79 to important role subscale, α = 0.77 to effort/improvement subscale, α = 0.82 to punishment for mistakes subscale, α = 0.86 to unequal recognition subscale, and α = 0.54 for the intra-team member rivalry subscale.

The Task and Ego Orientation in Sport Questionnaire (TEOSQ) (Duda & Nicholls, 1992) is a 13-item questionnaire designed to assess the goal orientation of athletes in a sports context (task and ego orientation) using a 5-point Likert scale. The Brazilian version of TEOSQ (Hirota et al., 2017), shows good internal consistency for both scales (task orientation α = 0.83, ego orientation α = 0.72), and maintained the same 13 items as the original version.

### Procedure

Permission to perform the cross-cultural adaptation and validation of PMCSQ-2 was obtained from one of the researchers of the original study (Newton et al., 2000). Subsequently, ethical approval was granted by the Research Ethics Committee of the Federal University of Paraná (CEP/SD–UFPR).

#### Translation/back-translation

Following the guidelines proposed by Beaton et al. (2007), the original questionnaire was sent to two independent bilingual translators for a complete translation of PMCSQ-2 from English into Portuguese (T1 and T2). These translations were analyzed by a committee of experts composed of two PhDs, one master’s degree, and a graduate student in physical education, who made a synthesis version (T12) of T1 and T2. The T12 version was sent to two other independent translators for back translation (Portuguese into English).

#### Semantic analysis

The translated version of the questionnaire (T12) was presented to the 34 athletes, who were requested to answer and identify any difficulties with comprehension.

#### Evidence based on test content

After translation and semantic analysis, the header and the items of the questionnaires were individually displayed on a Google Form® page to be evaluated in terms of “language clarity,” “practical pertinence,” and “theoretical relevance” by experts (Cassep-Borges et al., 2010).

Language clarity refers to how clear, understandable, and suitable the item is for the population to which the questionnaire is addressed. “Practical pertinence” analyzes whether each item has relevance to the questionnaire, as well as the importance of the item in the instrument for measuring a given construct. Theoretical relevance analyzes how much each item is associated with the underlying theory and aims to analyze whether the item is related to the construct. All evaluations were performed using a 5-point Likert scale ranging from 1 (very little) to 5 (very much). Below each item, there was a blank box where changes could be suggested; if the expert scored 3 or less on the Likert scale, the blank box had to be completed.

After these steps, the Brazilian version of the questionnaire was named PMCSQ-2BR for the remaining analyses.

#### Data collection

All subsequent data were collected during the 19th Paraná Open Games, held in the city of Campo Largo/PR between July 4 and 24, 2019, and the 32nd Paraná Youth Games, held in the city of Araucária/PR between July 22 and 28, 2019. The questionnaires were applied in the lodging or in the game centers upon the signing of the consent form by the athletes or their coaches when the athlete was under 18 years of age. The gap between the two collections required for test-retest reliability was 10–15 days.

### Data analysis

#### Evidence based on test content

To evaluate the level of agreement among experts on language clarity, practical pertinence, and theoretical relevance, the Content Validity Coefficient (CVC_C_) (Hernández-Nieto, 2002) was calculated for each item and the full questionnaire. To be accepted as valid, each item should score at least 0.80.

#### Evidence based on internal structure

CFA using WLSMV estimation and robust maximum likelihood (MLR) using R software v 3.6.1 (lavaan package v 0.6-4) was performed. Following the procedures conducted by the authors of the original instrument (Newton et al., 2000) six hypothetical models were tested in the CFA.

- *Model 01* is an orthogonal two-factor model with two first-order factors (task involving and ego involving).
- *Model 02* s an oblique two-factor model with two first-order factors (task involving and ego involving).
- *Model 03* is an orthogonal six-factor model with six first-order factors (cooperative learning, important role, effort/enjoyment, punishment for mistakes, unequal recognition, intrateam member rivalry).
- *Model 04* is an oblique six-factor model with six first-order factors (cooperative learning, important role, effort/enjoyment, punishment for mistakes, unequal recognition, intrateam member rivalry).
- *Model 05* is a hierarchical orthogonal model with two sets of orthogonal first-order factors (cooperative learning, important role, and effort/enjoyment in set 1, and punishment for mistakes, unequal recognition and intrateam member rivalry in set 2) and two orthogonal second-order factors (task-involving and ego-involving climate).
- *Model 06* is an oblique hierarchical model with six first-order factors (cooperative learning, important role, effort/enjoyment, punishment for mistakes, unequal recognition, intrateam member rivalry) and two second-order factors (task involving and ego involving climate).

To identify the model that best fit the data, various overall fit indices were analyzed. The goodness-of-fit index (GFI) and adjusted goodness-of-fit index (AGFI) were used to assess the goodness-of-fit indices; values greater than 0.90 were considered a good fit (Hu & Bentler, 1999). To identify the highest level of parsimony fit, we used the root mean square error of approximation (RMSEA), the parsimony normed fit index (PNFI), and parsimony goodness-of-fit index (PGFI). The RMSEA evaluates how well a particular model fits into the population (Brown, 2015), and values below 0.08 indicate a good fit, whereas values below 0.05 indicate a very good fit for the model (Hu & Bentler, 1999). For the PNFI and PGFI, values above 0.70 are considered acceptable because these indices hardly reach values above 0.90 (Newton et al., 2000). Absolute fit was assessed through the standardized root mean square residual (SRMR) and chi-square difference test (ΔX^2^), which was used to statistically compare the fit of two nested models. Values of SRMR less than 0.08 were considered acceptable and values less than 0.05, were considered very good (Hu & Bentler, 1999; Revesz et al., 2014).

As in the PMCSQ-2 construction study, the cutoff criteria were not interpreted rigidly by simply rejecting models that did not reach the parameters. Instead, the purpose was to evaluate several indices and thus identify the best fit model (Newton et al., 2000).

#### Internal consistency

Cronbach’s α (Cronbach, 1951) and Composite Reliability Coefficient (CR) were calculated to assess the internal consistency of the PMCSQ-2BR, and the commonly used cutting line of 0.70 for α and 0.60 for CR were considered as acceptable values.

#### Measurement invariance

Multiple-group CFA was performed to assess potential differences in factor loadings between the male and female participants. The difference in CFI (ΔCFI) was used to evaluate the invariance, where values > 0.01 among the different solutions (configural, metric, scalar, and strict) indicate a significant variation between groups.

#### Test–retest

To examine the test–retest reliability, we used the intraclass correlation coefficient (ICC; two-way mixed, consistency type, average score) (McGraw, 1996). The guidelines were followed as proposed by Cicchetti (1994), which considers an ICC of < 0.40 as poor, from 0.40 to 0.59 as fair, from 0.60 to 0.74 as good, and from 0.75 to 1.00 as excellent.

### Evidence based on relations to other variables

#### Discriminant evidence

Simple correlations between the different scales of the PMCSQ-2BR and TEOSQ were calculated in this step.

## Results

### Evidence based on test content

#### Semantic analysis

No item presented problems of interpretability for the athletes; thus, the version obtained in the synthesis of the translations for the next analyses was maintained.

#### Content validity coefficient

The content validity coefficients (CVC_C_) are listed in Table 1. For “practical pertinence” (*m* = 0.97, *s* = 0.02) and “theoretical relevance” (*m* = 0.97, *s* = 0.02), the complete questionnaire as well its 33 individual items presented values significantly greater than 0.80, whereas in the “language clarity” (*m* = 0.92, *s* = 0.05), only item 15 (“On this team, the coach yells at players for messing up”, CVCC = 0.75), included in the “punishment for mistakes” subscale, had a value below the cutoff criteria. After a review and consultation with athletes fluent in both languages (Portuguese and English), the specialists’ suggestions for changes in the item’s writing were accepted. The total CVC_C_ of the questionnaire was 0.96.

**Table 1:** Content validity coefficients (CVCc) of the 33 items and complete PMCSQ-2

Item	Language clarity	Practical pertinence	Theoretical relevance
1	0.80	0.92	0.95
2	0.92	0.92	0.92
3	0.90	0.97	0.97
4	0.87	0.97	0.98
5	0.95	0.98	1.00
6	0.87	0.95	0.95
7	0.92	0.97	0.98
8	0.85	0.95	0.93
9	0.93	0.97	0.97
10	0.98	0.98	0.98
11	0.87	1.00	0.97
12	0.85	0.95	0.97
13	0.95	0.98	0.97
14	0.80	0.97	0.97
15	0.75*	0.95	0.97
16	0.93	0.97	0.98
17	0.92	0.97	0.98
18	0.95	0.97	0.98
19	0.98	0.98	0.98
20	0.98	0.98	0.98
21	1.00	1.00	1.00
22	0.93	0.97	0.98
23	0.93	0.95	0.97
24	0.93	1.00	1.00
25	0.87	0.95	0.93
26	0.93	0.98	0.97
27	1.00	0.98	0.98
28	0.88	0.97	0.98
29	0.92	0.95	0.95
30	0.97	0.98	1.00
31	1.00	1.00	0.98
32	1.00	1.00	1.00
33	0.97	0.98	0.98
**PMCSQ-2BR**	**0.92**	**0.97**	**0.97**
Total	0.96		

*CVC_C_ < 0.80

### Evidence based on internal structure

#### Confirmatory factor analysis

Table 2 presents the overall fit indices for the six hypothesized models with two different estimators (WLSMV and MLR).

**Table 2:** Comparison of fit indices among the proposed models and different estimators (WLSMV/MLR)

Model/estimator	*X*^2^	df	Δ *X*^2^		GFI	AGFI	RMSEA (90% C.I.)		PNFI	PGFI	NNFI	SRMR	CFI
**1/WLSMV**	1104.47	495.00			0.88	0.86	0.05	(0.049–0.058)	0.73	0.77	0.74	0.10	0.76
**1/MLR**	1267.46	495.00			0.81	0.78	0.06	(0.057–0.064)	0.62	0.71	0.74	0.10	0.76
**2/WLSMV**	860.86	494.00	243.61	***	0.93	0.92	0.04	(0.037–0.046)	0.82	0.82	0.84	0.07	0.85
**2/MLR**	1231.90	494.00	35.56	***	0.81	0.78	0.06	(0.055–0.063)	0.63	0.71	0.76	0.07	0.77
**3/WLSMV**	2303.19	495.00			0.64	0.59	0.09	(0.089–0.096)	0.31	0.57	0.23	0.17	0.27
**3/MLR**	1811.00	495.00			0.76	0.73	0.08	(0.075–0.082)	0.49	0.67	0.57	0.17	0.60
**4/WLSMV**	828.42	480.00	1474.77	***	0.94	0.93	0.04	(0.036–0.046)	0.80	0.80	0.85	0.07	0.86
**4/MLR**	1128.55	480.00	682.46	***	0.84	0.81	0.06	(0.052–0.060)	0.64	0.71	0.78	0.07	0.80
**5/WLSMV**	1082.89	489.00			0.88	0.87	0.05	(0.049–0.058)	0.72	0.77	0.74	0.09	0.76
**5/MLR**	1196.83	489.00			0.83	0.80	0.06	(0.054–0.062)	0.64	0.72	0.77	0.09	0.78
**6/WLSMV**	830.55	488.00	252.35	***	0.94	0.93	0.04	(0.036–0.045)	0.81	0.81	0.85	0.07	0.86
**6/MLR**	1160.11	488.00	36.73	***	0.83	0.80	0.06	(0.053–0.061)	0.64	0.72	0.78	0.07	0.80

*X*^2^ chi-square, *df* degrees of freedom, *ΔX*^*2*^ difference between two nested models, *GFI* goodness-of-fit index, *AGFI* adjusted goodness-of-fit index, *RMSEA (90% C.I.)* root mean square error of approximation with 90% confidence interval, *PNFI* parsimony normed fit index, *PGFI* parsimony goodness-of-fit index, *NNFI* non-normed fit index, *SRMR* standardized root mean square residual, *CFI* comparative fit index

*** *p* < 0.001

Except for one model (Model 3), all other models showed good fit indices with the WLSMV estimator. Therefore, we follow the analyses with the results found with this estimator. Table 3 presents the overall fit indices for the six hypothesized models with the WLSMV estimator.

**Table 3:** Comparison of fit indices among the proposed models

Model	*X*^2^	df	Δ*X*^2^		GFI	AGFI	RMSEA (90% C.I.)		PNFI	PGFI	NNFI	SRMR	CFI
**1**	1104.47	495.00			0.88	0.86	0.05	(0.049–0.058)	0.73	0.77	0.74	0.10	0.76
**2**	860.86	494.00	243.61	***	0.93	0.92	0.04	(0.037–0.046)	0.82	0.82	0.84	0.07	0.85
**3**	2303.19	495.00			0.64	0.59	0.09	(0.089–0.096)	0.31	0.57	0.23	0.17	0.27
**4**	828.42	480.00	1474.77	***	0.94	0.93	0.04	(0.036–0.046)	0.80	0.80	0.85	0.07	0.86
**5**	1082.89	489.00			0.88	0.87	0.05	(0.049–0.058)	0.72	0.77	0.74	0.09	0.76
**6**	830.55	488.00	252.35	***	0.94	0.93	0.04	(0.036–0.045)	0.81	0.81	0.85	0.07	0.86

*X*^2^ chi-square, *df* degrees of freedom, *ΔX*^2^ difference between two nested models, *GFI* goodness-of-fit index, *AGFI* adjusted goodness-of-fit index, *RMSEA (90% C.I.)* root mean square error of approximation with 90% confidence interval, *PNFI* parsimony normed fit index, *PGFI* parsimony goodness-of-fit index, *NNFI* non-normed fit index, *SRMR* standardized root mean square residual, *CFI* comparative fit index

***p < 0.001

The results indicate that in all cases (two-factor models, six-factor models, and hierarchical models) the data fit better in oblique models (2, 4, 6) than in orthogonal models (1, 3, and 5). Even with Model 2 (*X*^2^ = 860.86, GFI = 0.93, AGFI = 0.92, RMSEA = 0.042, PNFI = 0.82, PGFI = 0.82, SRMR = 0.07, CFI = 0.85) and Model 4 (*X*^2^ = 828.42, GFI = 0.94, AGFI = 0.93, RMSEA = 0.041, PNFI = 0.80, PGFI = 0.80, SRMR = 0.07, CFI = 0.86) showing good fit indices, the subsequent analyses were conducted with Model 6 (*X*^2^ = 830.55, GFI = 0.94, AGFI = 0.93, RMSEA = 0.040, PNFI = 0.81, PGFI = 0.81, SRMR = 0.07, CFI = 0.86) because it is the final model of the original study, as well as more appropriate for the theory that supports it, and presented the best fit indices for this sample.

#### Assessment of individual parameter estimates

All individual factor loadings for Model 6 were between 0.25 and 0.80 (*t* value > 1.96) with positive and statistically significant (*t* value > 1.96) coefficients of determination (*R*^2^).

#### Interfactor correlation

Table 4 presents the observed correlation coefficients between the six subscales and their respective scales. Correlations of 0.85–0.91 were found between the task-involving scale and its subscales and 0.66–0.89 among the ego-involving scale and its subscales. All the aforementioned correlations were significantly related and in the expected direction.

**Table 4:** Correlation between subscales (1–6) and high-order scales (7–8)

Subscales	1		2		3		4		5		6		7		8
1.Cooperative learning	1.00														
2.Important role	0.67	***	1.00												
3.Effort/improvement	0.68	***	0.66	***	1.00										
4.Punishment for mistakes	− 0.17	*	− 0.21	***	− 0.08		1.00								
5.Unequal recognition	− 0.33	***	− 0.29	***	− 0.26	***	0.52	***	1.00						
6.Intra-team member rivalry	− 0.30	***	− 0.26	***	− 0.25	***	0.34	***	0.50	***	1.00				
7.Task-involving climate	0.85	***	0.87	***	0.91	***	− 0.17	*	− 0.32	***	− 0.30	***	1.00		
8.Ego-involving climate	− 0.31	***	− 0.30	***	− 0.22	***	0.78	***	0.89	***	0.66	***	− 0.30	***	1.00

***p < 0.001

*p < 0.05

#### Internal consistency

The Cronbach’s α value of the two high-order scales (task-involving α = 0.87; ego-involving α = 0.82) demonstrate good internal consistency for both. The CR coefficient (task-involving CR = 0.95; ego-involving CR = 0.92) shows excellent internal consistency for the two high-order scales of the PMCSQ-2BR. All three task-involving subscales (cooperative learning, important role, effort/improvement) and one ego-involving subscale (unequal recognition) had values between 0.70 and 0.76 for α and 0.72 and 0.78 for CR, showing acceptable internal consistency. Table 5 shows the results of Cronbach’s α and CR coefficient.

**Table 5:** Internal consistency estimation of PMCSQ-2BR and its scales

Subscales		Cronbach’s α	CR
Task-involving		0.87	0.95
	Cooperative learning	0.72	0.72
	Important role	0.70	0.70
	Effort/improvement	0.76	0.76
Ego-involving		0.82	0.92
	Punishment for mistakes	0.64	0.60
	Unequal recognition	0.76	0.78
	Intra-team member rivalry	0.47	0.46

#### Measurement invariance

Table 6 shows the results of the *X*^2^, df, CFI, and ΔCFI for the multiple-group CFA (measurement invariance). None of the ΔCFI values are greater than 0.01, which indicates that the factorial structure of Model 6 does not vary between groups (male and female).

**Table 6:** Test of measurement invariance of PMCSQ-2BR on male and female

Measurement invariance	*X*^2^		df	Δ*X*^2^	CFI	ΔCFI
Male (*n* = 349)	650.800	***	488		0.887	
Female (*n* = 152)	617.775	***	488		0.822	
Configural (equal form)	1268.539	***	976		0.862	
Metric (equal factor loading)	1278.062	***	1007	9.523	0.872	0.010
Scalar (equal indicator intercepts)	1318.353	***	1032	40.291	0.865	-0.007
Strict (equal indicator error variances)	1354.634	***	1065	36.28	0.863	-0.002

***p < 0.001

#### Intraclass correlation (test-retest)

The intraclass correlation coefficients, used to the test-retest analysis, shows good indices for “important role” (ICC = 0.70), “effort/improvement” (ICC = 0.68), “unequal recognition” (ICC = 0.71), and “intra-team member rivalry” (ICC = 0.70) subscales.

### Evidence based on relations to other variables

#### Discriminant evidence

From the discriminant evidence, simple correlations were calculated between the two high-order scales with their subscales and the two dimensions of the TEOSQ (Hirota et al., 2017) (Table 7).

**Table 7:** Correlations between the six scales of PMCSQ-2BR and two scales of TEOSQ

	Goal orientation (TESOQ)			
Perceived Motivational Climate (PMCSQ-2BR)	Task orientation		Ego orientation	
Cooperative learning	0.39	***	− 0.07	
Important role	0.40	***	− 0.04	
Effort/improvement	0.50	***	− 0.05	
**Task-involving**	0.50	***	− 0.06	
Punishment for mistakes	− 0.04		0.07	
Unequal recognition	− 0.15		0.24	***
Intra-team member rivalry	− 0.23	***	0.23	***
**Ego-involving**	− 0.13	*	0.22	***

***p < 0.001

*p < 0.05

The task-involving climate scale and its three subscales were significant and positively correlated with the task orientation dimension of the TEOSQ (cooperative learning = 0.39, important role = 0.40, effort/improvement = 0.50, task-involving = 0.50) similar to the ego-involving climate scale and two of its subscales with the ego orientation dimension (unequal recognition = 0.24, intrateam member rivalry = 0.23, ego-involving = 0.22).

## Discussion

The first source of validity evidence analyzed was based on the test content, which allows the demonstration of the relationship between the content of the instrument (themes, words, and format of items, tasks, or questions in a test) and the construct to be measured. This step presented excellent CVC_C_ values for the entire questionnaire (language clarity, practical pertinence, theoretical relevance), thus reducing the chances of moving on to the next validation steps with failures such as unequally measured dimensions, items assessing different constructs from those originally proposed by the instrument, and lack of items that evaluate the construct (Haynes et al., 1995; Hernández-Nieto, 2002). The marginal value of CVC_C_ obtained by item 15 in “language clarity” may have been caused by the difficulty in translating the term “messing up” into Portuguese (“On this team, the coach yells at players for messing up”). Although all the athletes participating in the semantic analysis expressed no difficulty with the item, we chose to follow the suggestion of change proposed by the specialists because this would not change the meaning of the phrase (“On this team, the coach yells at players for ‘making serious mistakes’”). Because neither the original study nor any other validation of the PMCSQ-2 has been conducted or presented this source of evidence, it is impossible to compare the results.

The second source of validity evidence was based on the internal structure of the questionnaire and was primordially evaluated by the CFA conducted in the six different models proposed by Newton et al. (2000). The results corroborate those of other studies, supporting Model 6 (oblique hierarchical) as the most fitted to the questionnaire. The hypothesis that made use of the WSLMV estimator in CFA providing better fit indices than the ML estimator was confirmed because only one SRMR was not sensitive to the estimation method, and all the other fit indices showed better results in the WSLMV estimator. When the questionnaire was developed (Newton et al., 2000), the authors performed the CFA with variations in Models 2, 4, and 6 (error covariance between items, cross-loaded items, excluded items, and excluded subscales); however, once the PMCSQ-2 was validated and used in several studies around the world without these changes, this step was not shown to be necessary in our study.

The fact that all items of the questionnaire presented positive and significant values of factor loading and coefficient of determination suggests that each item was adequately measured and contributed significantly to the assessment of its respective construct. Likewise, the six subscales (cooperative learning, important role, effort/improvement, punishment for mistakes, unequal recognition, intra-team member rivalry) showed a coefficient of correlation in the expected direction and with a p-value lower than 0.001, suggesting that all six subscales were correctly specified on a high-order factor.

The two high-order scales, exhibited high internal consistency. All three task-involving subscales and one ego-involving scale (unequal recognition) showed acceptable to good internal consistency, whereas the “punishment for mistakes” subscale presented a questionable score for α and acceptable for CR. However, the internal consistency of the “intra-team member rivalry” was confirmed to be problematic (α = 0.47; CR = 0.46), as in the study of Newton et al. (2000). The use of the CR calculation is justified by the fact that this is a more robust equation than Cronbach’s alpha, since it is sensitive to the factorial load of each item in its factor, as well as to the number of items used in each scale. Thus, although there are indications of a cutoff line (CC > 0.60), its interpretation should not be made so directly (Fornell; Larcker, 1981). In a simulation carried out to detect how sensitive to the number of items the CR value could be, Valentini and Damásio (2016) identified that, with the same average standardized factor loading, the results obtained for CR vary significantly in instruments with 5, 10, or 30 items, being the value higher as the number of items increases (e.g., the CC values for instruments with average factor loadings of 0.40 vary from 0.40–0.50, in instruments with five items, to 0.80–0.90, in instruments with 30 items).

These findings may justify the low values obtained for the subscale team member rivalry. A direct reading of the results would indicate low internal consistency of the subscale, however, because it is a dimension with only three items and an average factor loading of 0.46, this result could be reinterpreted as satisfactory.

The original authors of PMCSQ-2 argue that the fact that their sample includes only female athletes may be responsible for the poor internal consistency because coaches and female athletes may be less likely to provide and participate fully in rivalrous training drills and interactions (Newton et al., 2000). However, this hypothesis could not be confirmed in our research, considering that our sample contained both male (69.66%) and female (30.34%) athletes; yet, this first-order factor was found to have poor internal consistency.

The few items in this subscale (only three) has already been raised by other researchers (Newton et al., 2000; Revesz et al., 2014) as an indicator of its inconsistency; however, some other aspects may influence these results: (a) the application of the questionnaire usually occurs in training sessions or competitions in which the athletes are involved. In these conditions, the teammates usually remain close to each other, which can influence the answers of some participants because this subscale evaluates how much the rivalry between team members is encouraged by the coach. (b) The drafting of item 12 (“On this team, players are encouraged to outplay the other players”) may have confused participants’ responses because in Brazilian Portuguese, it may not be clear that the “other players” are actually their teammates.

In addition, the decrease in CFI for the female’s group in the “measurement invariance” may have been caused by the difference in the sample size (349 males and 152 females), given that, in many aspects the CFA results can be influenced by the numbers of subjects (Brown, 2015). However, none of the variation of the CFI (ΔCFI) was greater than 0.010 in the subsequent analyses, which indicates that the questionnaire is invariant between groups (male/female). These findings also support the rebuttal of the hypothesis that female athletes could have differences in the assessment of some construct by the PMCSQ-2.

The hypothesis that a positive relationship would arise between perceptions of a task-involving motivational climate, assessed through PMCSQ-2BR, and task orientation, measured by TEOSQ, was supported (*r* = 0.50, *p* < 0.001). The same occurred with “cooperative learning,” “important role,” “effort/improvement,” and the task orientation scale; all three were significant and in the expected direction (*r* = 0.39, *r* = 0.40, *r* = 0.50, *p* < 0.001, respectively).

Moreover, the hypothesis that the correlations would be positive between the perception of an ego-involving motivational climate (and its second-order scales) and ego orientation was partially supported. “Unequal recognition” (*r* = 0.24, *p* < 0.001), “intra-team member rivalry” (*r* = 0.23, *p* < 0.001), and “ego involving” (*r* = 0.22, *p* < 0.001) were minimal although significant, which was related to the ego orientation scale of the TEOSQ. However, the perception of being punished for mistakes appears to be unrelated to ego-oriented motivation. Additionally, the perception of an ego-involving motivational climate (*r* = − 0.13, *p* < 0.05) and that rivalry among teammates is reinforced by the coach (*r* = − 0.23, *p* < 0.001) seems to be negatively correlated with task-oriented motivation. These results suggest that the perception of a task-involving coach-created motivational climate is more strongly correlated with the existence of task-oriented athletes than the perception of an ego-involving coach-created motivational climate is with ego-oriented athletes. Even if both are positive in their magnitude.

These findings corroborate the results of similar studies (Balaguer et al., 1997; Selfriz et al., 1992) and the original framework of AGT (Ames, 1992; Dweck, 1986; Nicholls, 1984), which indicates that the motivational climate created by the coach can influence the goal orientation of an athlete.

### Limitations and future research directions

Although this study provides an important addition to the research on the perception of the coach-created motivational climate, the limitations should also be acknowledged. The participation of athletes only representing teams from the Paraná State, along with the inclusion of five interdependent sports, may not provide generalizability of the results to other sports and locations. We suggest increasing the number of sports examined by the instrument, as well as more states in Brazil.

We suggest caution in the utilization and interpretation of the “intra-team member rivalry” subscale by its own, since its internal consistency values were below the proposed cut-off line.

Additional future research could include a comparison of the perceived motivational climate with other psychological constructs that are relevant to athletes’ sport development and performance.

## Conclusion

This study aimed to obtain sources of validity evidence and reliability of the Brazilian version of the PMCSQ-2BR, an instrument that evaluates the athletes’ perception of the coach-created motivational climate in sports context.

The results of the analyses support the multidimensional hierarchical structure of the PMCSQ-2 in the Brazilian sports context. Model 6 (oblique hierarchical model with six first-order factors and two second-order factors) had the best fit (*X*^2^ = 830.55, GFI = 0.94, AGFI = 0.93, RMSEA = 0.040, PNFI = 0.81, PGFI = 0.81, SRMR = 0.07, CFI = 0.86), as in the original study (Newton et al., 2000), and followed the notion that the motivational climate is multifaceted (Ames, 1992; Walling et al., 1993). In addition, the hypothesis that the WLSMV estimator would be more adequate for the CFA of the PMCSQ-2BR was confirmed.

The tests presented in this study confirm the high internal consistency of the two high-order scales (task-involving and ego-involving climate). However, the “intra-team member rivalry” scale needs to be reviewed, especially item 12, where we suggest changing its writing.

Further studies should be performed in the Brazilian sports context, correlating the perception of the motivational climate created by the coach with other variables relevant to the performance and health of athletes, allowing them to be compared with similar studies performed in other countries (Curran et al., 2015; García-Calvo et al., 2014; Ruiz-Sánchez et al., 2017).

## Data Availability

All data generated or analyzed during this study are included in this published article.

## Abbreviations

AGFI: Adjusted Goodness-of-Fit Index
AGT: Achievement Goal Theory
CFA: Confirmatory factor analysis
CFI: Comparative Fit Index
CNPq: National Council for Scientific and Technological Development
CR: Composite reliability coefficient
CVCc: Content validity coefficient
GFI: Goodness-of-Fit Index
ICC: Intraclass correlation coefficient
ML: Maximum likelihood
MLR: Robust maximum likelihood
NNFI: Non-normed Fit Index
PFGI: Parsimony Goodness-of-Fit Index
PMCSQ-2: Perceived Motivational Climate in Sports Questionnaire–2
PNFI: Parsimony Normed Fit index
RMSEA: Root mean square error of approximation
SRMR: Standardized root mean square residual
TEOSQ: Task and Ego Orientation in Sport Questionnaire
WLSMV: Weighted least square mean and variance adjusted
